# miR‐148a suppresses inflammation in lipopolysaccharide‐induced endometritis

**DOI:** 10.1111/jcmm.14744

**Published:** 2019-11-22

**Authors:** Kangfeng Jiang, Jing Yang, Chao Yang, Tao Zhang, Aftab Shaukat, Xiaoyan Yang, Ailing Dai, Haichong Wu, Ganzhen Deng

**Affiliations:** ^1^ Department of Clinical Veterinary Medicine College of Veterinary Medicine Huazhong Agricultural University Wuhan China; ^2^ Fujian Provincial Key Laboratory for the Prevention and Control of Animal Infectious Diseases and Biotechnology Longyan China; ^3^ State Key Laboratory of Agricultural Microbiology College of Veterinary Medicine Huazhong Agricultural University Wuhan China; ^4^ College of Life Sciences of Longyan University Longyan China

**Keywords:** endometritis, inflammation, miR‐148a, NF‐κB, TLR4

## Abstract

Endometritis is a postnatal reproductive disorder disease, which leads to great economic losses for the modern dairy industry. Emerging evidence indicates that microRNAs (miRNAs) play a pivotal role in a variety of diseases and have been identified as critical regulators of the innate immune response. Recent miRNome profile analysis revealed an altered expression level of miR‐148a in cows with endometritis. Therefore, the present study aims to investigate the regulatory role of miR‐148a in the innate immune response involved in endometritis and estimate its potential therapeutic value. Here, we found that miR‐148a expression in lipopolysaccharide (LPS)‐stimulated endometrial epithelial cells was significantly decreased**.** Our results also showed that overexpression of miR‐148a using agomiR markedly reduced the production of pro‐inflammatory cytokines, such as IL‐1β and TNF‐α. Moreover, overexpression of miR‐148a also suppressed NF‐κB p65 activation by targeting the TLR4‐mediated pathway. Subsequently, we further verified that miR‐148a repressed TLR4 expression by binding to the 3′‐UTR of TLR4 mRNA. Additionally, an experimental mouse endometritis model was employed to evaluate the therapeutic value of miR‐148a. In vivo studies suggested that up‐regulation of miR‐148a alleviated the inflammatory conditions in the uterus as evidenced by H&E staining, qPCR and Western blot assays, while inhibition of miR‐148a had inverse effects. Collectively, pharmacologic stabilization of miR‐148a represents a novel therapy for endometritis and other inflammation‐related diseases.

## 1 INTRODUCTION

Uterine function is usually compromised in the postpartum dairy cow by intrauterine bacterial infections,^1^, ^2^ and bacterial infections are believed to be closely correlated with clinical and subclinical endometritis.^3^ Clinical endometritis is often featured by the presence of purulent or mucopurulent secretions in the vagina 21 days or more postpartum; by contrast, subclinical endometritis is defined as the inflammation of the endometrium in the absence of clinical symptoms.^4^ Cow endometritis often causes repeated implantation failure and infertility, thus leading to serious economic losses for the modern dairy industry.^5^, ^6^ The common pathogenic bacteria associated with endometritis are *Escherichia coli* (*E coli*) and other Gram‐negative bacteria.^7^ *Escherichia coli* generally infects the endometrium prior to other pathogens, paving the way for other pathogens to cause endometrial injury and disturbing the ovarian cycle.^8^ The clinical application of antibiotics in cow endometritis has achieved certain treatment effects, but new therapeutic strategies for endometritis are needed, as the problems of the bacterial resistances and drug residues remain unresolved.

In the reproductive tract, the initial defence of endometrium against invasive bacteria depends on the innate immune system.^9^ Once the innate immune system is activated, endometrial cells secrete a large number of cytokines and chemokines, which can recruit neutrophils and macrophages to eliminate pathogens.^10^ However, excessive inflammatory reactions can aggravate tissue damage to endometrium and even cause systemic inflammation. At the same time, these abnormal changes in the endometrium also interfere with endocrine function and are incompatible with sperm transport and embryo implantation.^3^ For instance, exposure to an inflammatory endometrial environment can decrease the number of trophoblasts.^11^ It has been well established that toll‐like receptors (TLRs) are crucial for the innate immune response against microbial invasion.^12^ TLRs are widely expressed by various immune cell such as macrophages, which mediate the activation of intracellular signalling pathways.^13^ In addition, they are also expressed by the cells of the reproductive system, including endometrial epithelial cells^14^ and ovarian follicle granulosa cells.^15^ An increasing body of studies suggest that TLR4 recognizes specific pathogen‐associated molecular patterns (PAMPs), such as lipopolysaccharide (LPS) from *E coli*, thus triggering the activation of NF‐κB pathway.^16^ Indeed, TLR4‐dependent signalling mechanisms of the innate immune system are essential for the response to LPS by epithelial cells of the bovine endometrium.^14^ NF‐κB, a crucial nuclear transcription factors, has been reported to directly regulate the production of pro‐inflammatory mediators and is correlated with the pathogenesis of endometritis.^17^, ^18^

MicroRNAs (miRNAs) are a class of small noncoding single‐stranded RNA molecules, which are usually 20‐22 nucleotides (nts) in length.^19^ Mature miRNAs bind to the 3′‐untranslated regions (UTRs) or coding sequences of specific target mRNAs, thereby resulting in their translational repression or degradation.^20^ In recent decades, some miRNAs, which are primarily highly conservative, have rawn more and more attention due to their vital function in a wide range of basic biological processes.^21^, ^22^ Although miRNAs are closely correlated with tumorigenesis and progression in various cancers, they also occupy an essential place in the progress of innate immune response.^23^ For example, miR‐146 is strongly induced in LPS‐challenged THP‐1 cells and negatively regulates the inflammatory response by inhibiting TNFR‐associated factor 6 (TRAF6) and IL‐1R‐associated kinase‐1 (IRAK1), two key factors of the TLR4 signalling.^24^ miR‐92a blunts TLR‐elicited inflammatory response in macrophages by directly inhibiting mitogen‐activated protein kinase kinase 4 (MKK4).^25^ Importantly, aberrant expression of miRNAs has also been reported to be associated with the disturbance of bovine reproductive function due to pathogenic infection.^26^ A recent report from Ibrahim et al^26^ revealed that bacterial LPS challenge led to differential expression of miRNAs in bovine oviductal cells, which leads to a suboptimal environment for embryo development. Recent miRNome profile analysis indicated that a great number of uterine miRNAs are involved in the establishment and progression of endometritis; amongst them, miR‐148a, a highly conservative miRNA in mammals, showed a differential expression pattern in cows with endometritis.^27^ miR‐148a has been shown to be an important modulator of inflammatory diseases, such as colitis.^28^ However, very little is known about miR‐148a's role in the innate immune response during cow endometritis. In the present study, using endometrial epithelial cells as an in vitro inflammatory model, we investigated the possible role of miR‐148a in the innate immune response implicated in the pathogenesis of endometritis. Meanwhile, a mouse model of LPS‐induced endometritis was also employed to determine whether miR‐148a functions as a potential therapeutic target for treating endometritis.

## 2 MATERIALS AND METHODS

### 2.1 Cell culture and treatment

The primary bovine endometrial epithelial cells (bEECs) were isolated and cultured as previously described.^29^ Briefly, the uterus tissues were aseptically obtained from healthy Holstein cows and washed 3 times with pre‐cooled PBS contained with 100 IU/mL penicillin and streptomycin. After digestion with 0.05% collagenase I (Sigma), the cells were isolated and cultured in DMEM/F12 (HyClone) containing 15% foetal bovine serum (FBS; Sigma). The primary endometrial epithelial cells were identified using specific antibodies against cytokeratin 18. The bovine endometrial epithelial cell line BEND cells and the human embryonic kidney cell line HEK293T cells were purchased from American Type Culture Collection (ATCC). BEND cells were cultured in DMEM/F‐12 supplemented with 10% FBS. HEK293T cells were grown in RPMI 1640 (HyClone) with 10% FBS.

To investigate the functional role of miR‐148a in the innate immune response, epithelial cells were treated with LPS (Sigma) to mimic an in vitro inflammatory model. After the indicated treatments, the cells were prepared for further experiments.

### 2.2 Cell transfection

miR‐148a agomiR or antagomiR, TLR4 siRNAs (si‐TLR4) and corresponding negative controls (NC) were designed and synthesized by GenePharma. The cells were transfected with miR‐148a agomiR, antagomiR or si‐TLR4 using Lipofectamine 2000 (Invitrogen) according to the manufacturer's instructions. The transfection efficiency was evaluated by qPCR assay.

### 2.3 Plasmids construction and luciferase reporter assay

The possible binding sites between miR‐148a and TLR4 were predicted using the bioinformatics software programs TargetScan 7.2. The wild‐type TLR4 3′‐UTR luciferase reporter vectors were constructed by PCR amplifying the TLR4 mRNA 3′‐UTR sequences containing the binding sites and cloning it into the psiCHECK^TM^‐2 vector (Promega). The binding sites of miR‐148a were mutated in the TLR4 3′‐UTR Mut vectors. For the luciferase reporter assay, HEK293T cells were transiently cotransfected with luciferase reporter vectors and miR‐148a agomiR or the corresponding negative controls, respectively. After 24 hours, the cells were prepared for the luciferase assay using the Dual‐Luciferase Reporter Assay System (Promega).

### 2.4 Animals and treatment

BALB/c mice (8 weeks old) were provided by the Experimental Animal Center of Huazhong Agricultural University (Wuhan, China). All mice were housed at a constant temperature (23‐25°C) and a relative humidity (40%‐80%) with a 12‐hour light/dark cycle and provided free food and water. All animal experiments in this study were carried out according to the NIH guidelines and approved by the Ethical Committee on Animal Research at Huazhong Agricultural University.

To examine whether miR‐148a acts as a possible molecular target for treating endometritis, a mouse model of LPS‐induced endometritis was employed in the present study. The murine model of endometritis was established as previously described.^30^ Briefly, the mice were infused equal volumes of LPS (1 mg/kg) on each side of the uterus for 24 hours, and the control mice received equal amounts of sterile phosphate‐buffered saline (PBS). To transiently increase or decrease the miR‐148a expression, mice received on three consecutive days, intraperitoneal injections of synthetic miR‐148a agomiR or antagomiR before LPS infusion. After indicated treatments, the mice were euthanized, and then, the uterine tissues were harvested and stored at −80°C for subsequent analysis.

### 2.5 Histological analysis

Tissue samples were fixed overnight in 4% paraformaldehyde, embedded in paraffin and then sliced into 4‐µm‐thick sections. The sections were stained with haematoxylin and eosin (H&E), and the histopathological changes were subsequently examined under a light microscope.

### 2.6 Cytokine assays

The levels of TNF‐α and IL‐1β were detected with ELISA kits, which were used following the producer's directions. The optical density (OD) value was read at 450 nm with a microplate reader (Thermo Scientific).

### 2.7 qPCR and Western blot

qPCR and Western blot were carried out according to the manufacturer's instructions as previously reported.^31^ qPCR was performed using SYBR Premix DimerEraser (Takara) on a 7900HT system. The relative expression of miR‐148a and specific mRNAs was normalized against internal controls (U6 or GAPDH) using the 2^−ΔΔCt^ method. For Western blot analysis, the density of each protein band was normalized to that of β‐actin band and quantified using the IPP 6.0 software.

### 2.8 Immunofluorescence staining

At the end of the treatment, the tissue sections or formalin‐fixed cells were incubated overnight at 4°C with special primary antibodies after the blocking of non‐specific protein binding by 10% bovine serum albumin, followed by incubation with FITC‐labelled secondary antibodies for 1 hour at 37°C and then stained by DAPI fluorescence dye for 15 minutes. Fluorescent images were captured with an inverted fluorescence microscope.

### 2.9 Statistical analysis

All the data were managed using GraphPad Prism 6.0 software and shown as the means ± SEM. The statistical significance was determined by an unpaired Student's *t* test for the comparison of two groups or one‐way analysis of variance (ANOVA) with Dunnett's test for multiple comparisons. Values of *P* < .05 were regarded as statistically significant.

## 3 RESULTS

### 3.1 Reduced expression of miR‐148a following LPS stimulation in vitro

**Figure 1:**
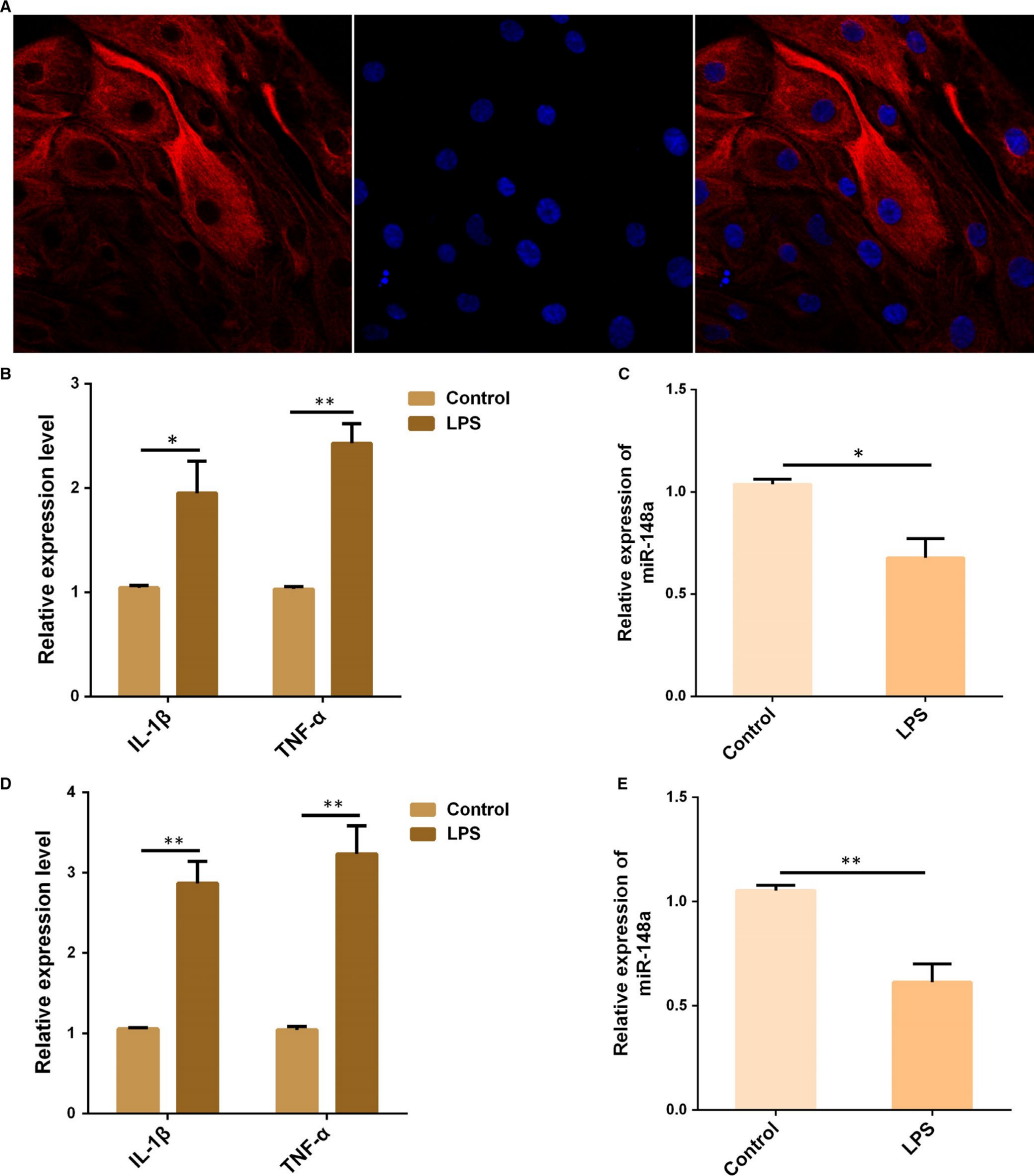
LPS reduced miR‐148a expression in vitro. A, Primary bovine endometrial epithelial cells (bEECs) were pre‐treated with fluorescent dyes to identify their integrity and purity. Epithelial‐specific marker cytokeratin 18 was labelled with a red fluorescence, and the cell nuclei were stained by DAPI (blue). B, bEECs were stimulated by LPS for 12 h, and then, the expression of IL‐1β and TNF‐α was detected by qPCR. C, The miR‐148a expression in bEECs was measured by qPCR. D, BEND cells were treated as (B), and the expression of IL‐1β and TNF‐α was detected by qPCR. E, The miR‐148a expression in BEND cells was measured by qPCR. Data are presented as the mean ± SEM. *P < .05; **P < .01

In order to uncover whether miR‐148a is implicated in the innate immune reaction of the endometrium, primary bovine endometrial epithelial cells (bEECs) were isolated (Figure 1A) and stimulated with LPS for 12 hours, and the pro‐inflammatory cytokines IL‐1β and TNF‐α were noticeably up‐regulated (Figure 1B). Subsequently, we measured the miR‐148a expression in bEECs following LPS stimulation. As displayed in Figure 1C, miR‐148a expression was remarkably down‐regulated upon LPS stimulation. Moreover, these findings were further verified in the bovine endometrial cell line BEND cells (Figure 1D,E). These results imply that miR‐148a is involved in the LPS‐triggered innate immune response.

### 3.2 miR‐148a decreases LPS‐induced production of pro‐inflammatory cytokines

**Figure 2:**
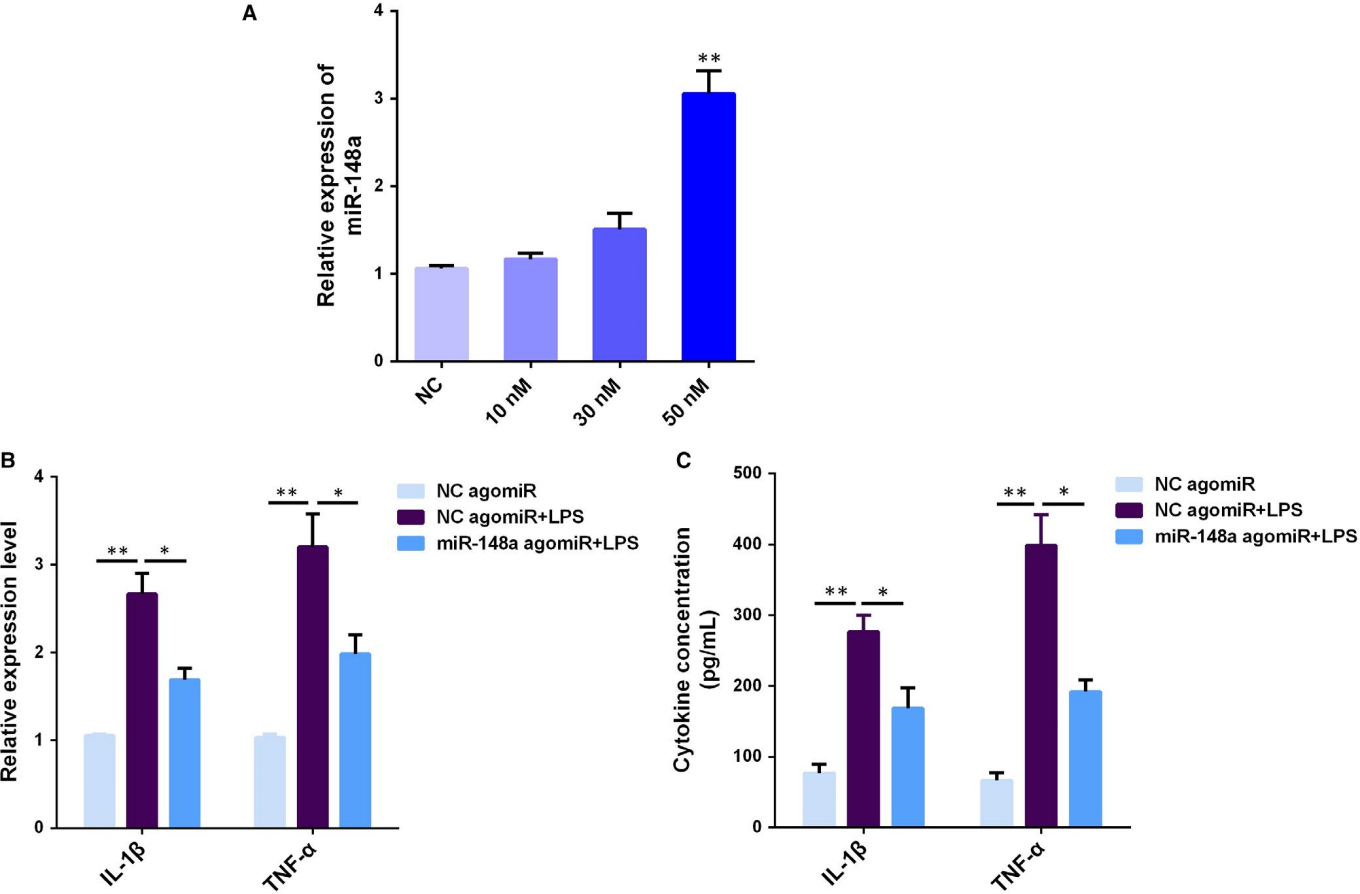
miR‐148a decreases LPS‐induced pro‐inflammatory cytokines production. (A) BEND cells were transfected with miR‐148a agomiR at different concentrations (0‐50 nM) for 24 h, and qPCR was used to measure the miR‐148a expression. BEND cells were transfected with miR‐148a agomiR, followed by 12 h of exposure to LPS, and the IL‐1β and TNF‐α production was detected by qPCR (B) and ELISA (C). Data are presented as the mean ± SEM. *P < .05; **P < .01

To ferret out the specific role of miR‐148a in the inflammatory response induced by LPS, BEND cells were transfected with miR‐148a agomiR for 24 hours (Figure 2A), followed by 12 hours of exposure to LPS, and then, the pro‐inflammatory cytokines IL‐1β and TNF‐α were measured by qPCR and ELISA. As shown in Figure 2B,C, overexpression of miR‐148a using agomiR significantly decreased LPS‐induced production of IL‐1β and TNF‐α. These data indicate that miR‐148a may possess anti‐inflammatory properties in the LPS‐mediated inflammatory response.

### 3.3 miR‐148a suppresses LPS‐induced activation of NF‐κB pathway

**Figure 3:**
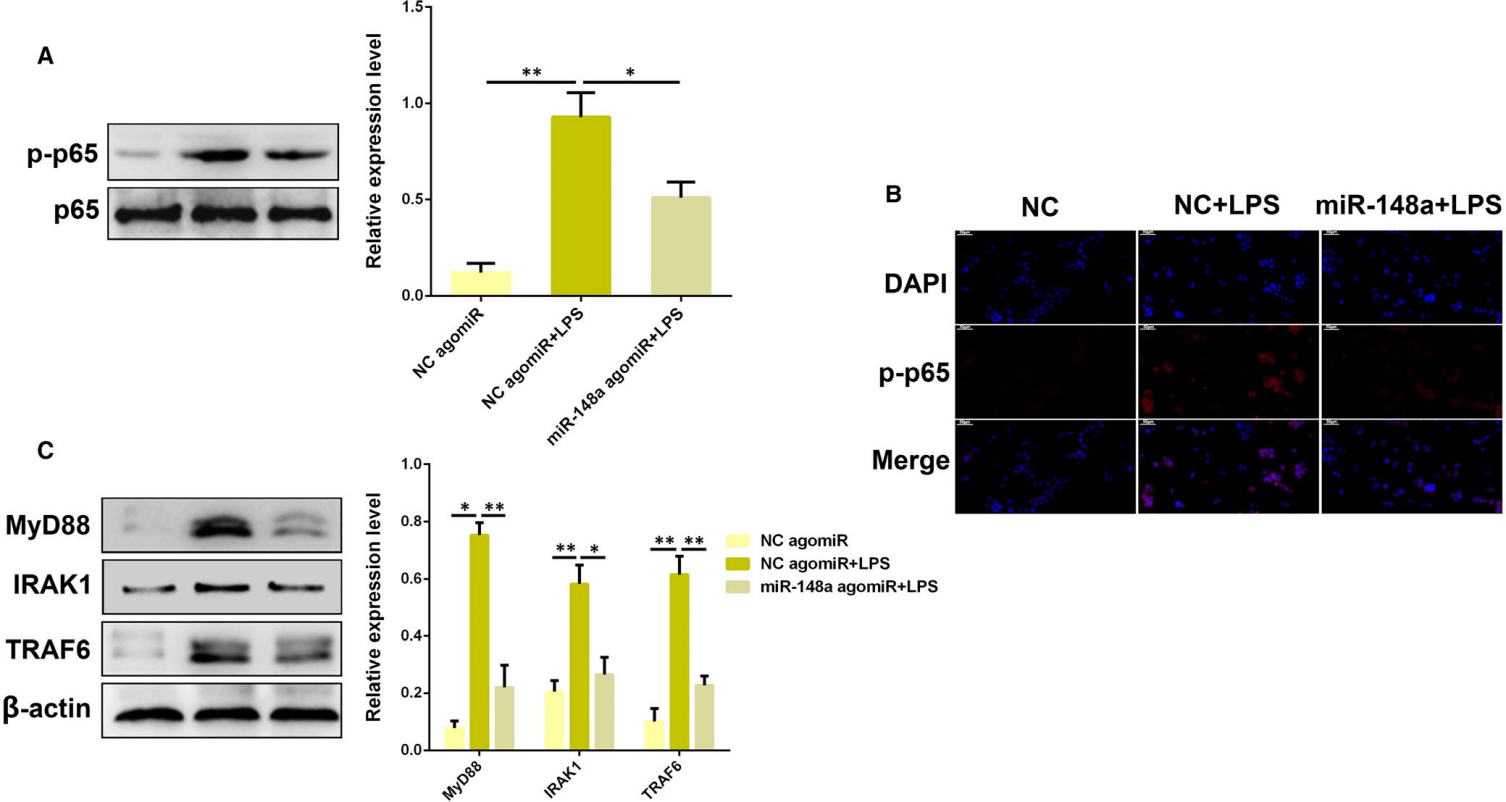
miR‐148a suppresses LPS‐induced activation of NF‐κB pathway. A, BEND cells were transfected with miR‐148a agomiR or the negative control (NC agomiR), followed by 12 h of exposure to LPS, and the protein level of NF‐κB p65 was determined by Western blotting. B, Translocation of the NF‐κB p65 subunit from the cytoplasm into the nucleus was assessed by immunofluorescence staining, Blue spots represent cell nuclei, and red spots indicate p‐p65 staining. C, The protein levels of upstream molecules (MyD88, IRAK1 and TRAF6) of NF‐κB pathway were detected by Western blotting. Data are presented as the mean ± SEM. *P < .05; **P < .01

As shown in Figure 3A, LPS‐induced NF‐κB activation, as demonstrated by increased phosphorylateion level of NF‐κB p65, which was significantly reversed by overexpression of miR‐148a. Meanwhile, immunofluorescence analysis also confirmed that miR‐148a suppressed the activation of NF‐κB p65 induced by LPS (Figure 3B). Typically, miRNAs function by directly binding to the 3′‐UTR of target gene mRNAs. Thus, we determined the effects of miR‐148a on the upstream molecules of NF‐κB pathway so as to find out the specific molecular targets of miR‐148a. The protein levels of MyD88, IRAK1 and TRAF6 that were up‐regulated by LPS were observably inhibited after miR‐148a overexpression, as detected by Western blot analysis (Figure 3C). These results demonstrate that miR‐148a impairs LPS‐triggered inflammatory response by restraining the activation of NF‐κB pathway.

### 3.4 TLR4 is a molecular target of miR‐148a

**Figure 4:**
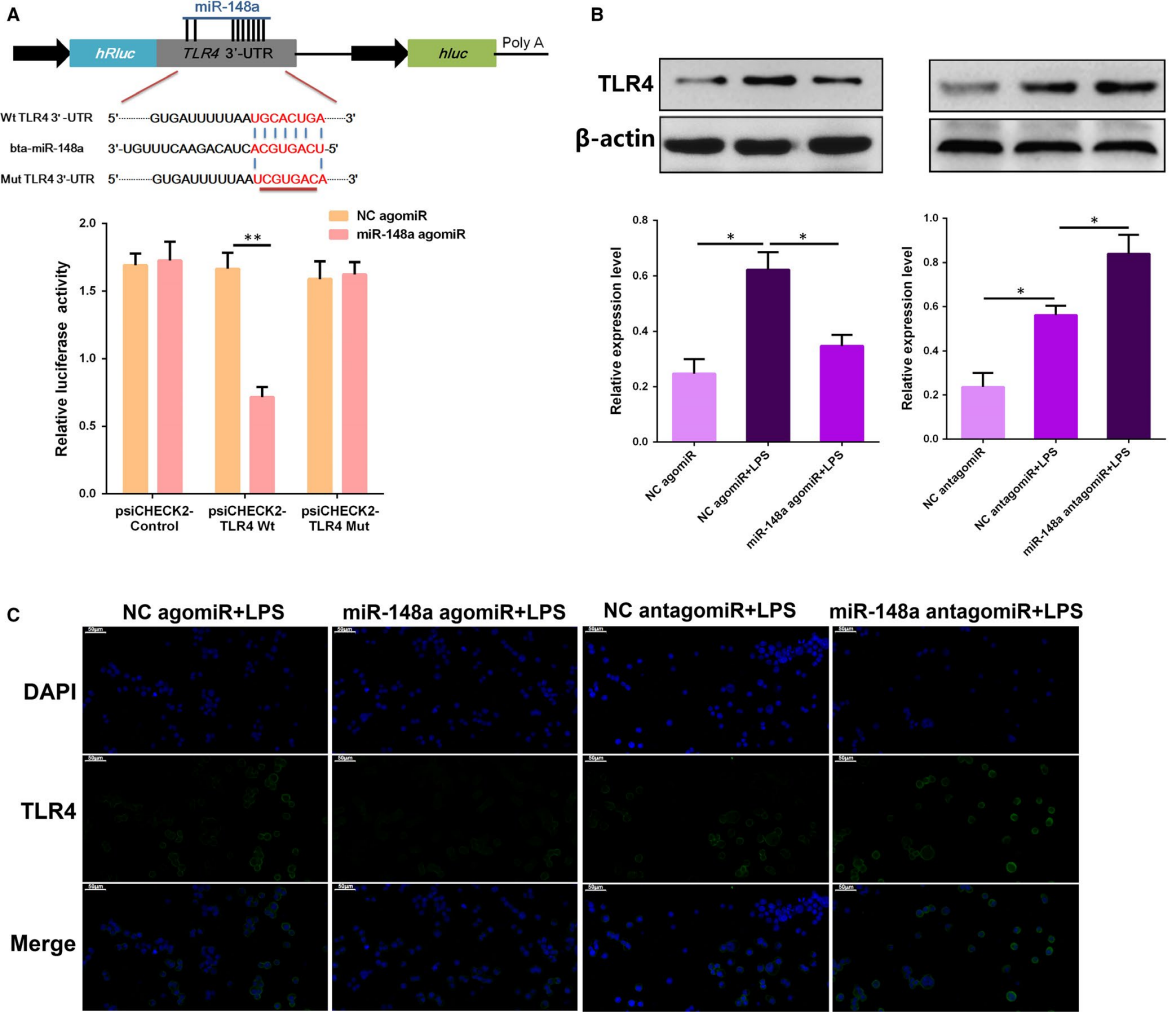
TLR4 is a molecular target of miR‐148a. A, The schematic graph displayed pairing relationship between miR‐148a and TLR4 mRNA 3′‐UTR. The dual‐luciferase reporter assay was carried out in HEK293T cells. Cells were cotransfected with the wild‐ or mutant‐type reporter vectors, as well as miR‐148a agomiR or the negative control (NC agomiR). The ratio of Renilla activity/Firefly activity represents luciferase activity. B, BEND cells were transfected with miR‐148a agomiR, miR‐148a antagomiR, or the corresponding negative controls (NC agomiR, NC antagomiR), and then, the TLR4 protein levels were measured by Western blotting. C, Immunofluorescence staining of TLR4 in BEND cells transfected with miR‐148a agomiR or miR‐148a antagomiR. Data are presented as the mean ± SEM. *P < .05; **P < .01

Intriguingly, the bioinformatics software program TargetScan 7.2 revealed that the 3′‐UTR of TLR4 mRNA contains a complementary site for the seed region of miR‐148a (Figure 4A). To verify the binding site, a luciferase reporter assay was performed in subsequent studies. As expected, we observed that miR‐148a agomiR significantly reduced the luciferase activity for the wild‐type 3′‐UTR of TLR4 mRNA but showed no suppression effect for the mutated 3′‐UTR of TLR4 mRNA (Figure 4A), suggesting that miR‐148a is able to bind to the 3′‐UTR of TLR4 mRNA. To further identify whether TLR4 is a molecular target of miR‐148a, a gain or loss of function approach was applied in subsequent studies. As shown in Figure 4B, the TLR4 protein level that was up‐regulated by LPS was dramatically repressed by overexpression of miR‐148a, whereas inhibition of miR‐148a increased TLR4 expression. Similar observations were also obtained by immunofluorescence analysis. Besides, we also examined the effect of miR‐148a alone on TLR4 expression in the absence of LPS, and we found that the TLR4 level decreased when miR‐148a was overexpressed, but increased when miR‐148a was knocked down (Figure S1). Overall, these results imply that miR‐148a negatively regulates TLR4 expression through targeting the 3′‐UTR of TLR4 mRNA.

### 3.5 Knockdown of TLR4 attenuates LPS‐induced inflammation

**Figure 5:**
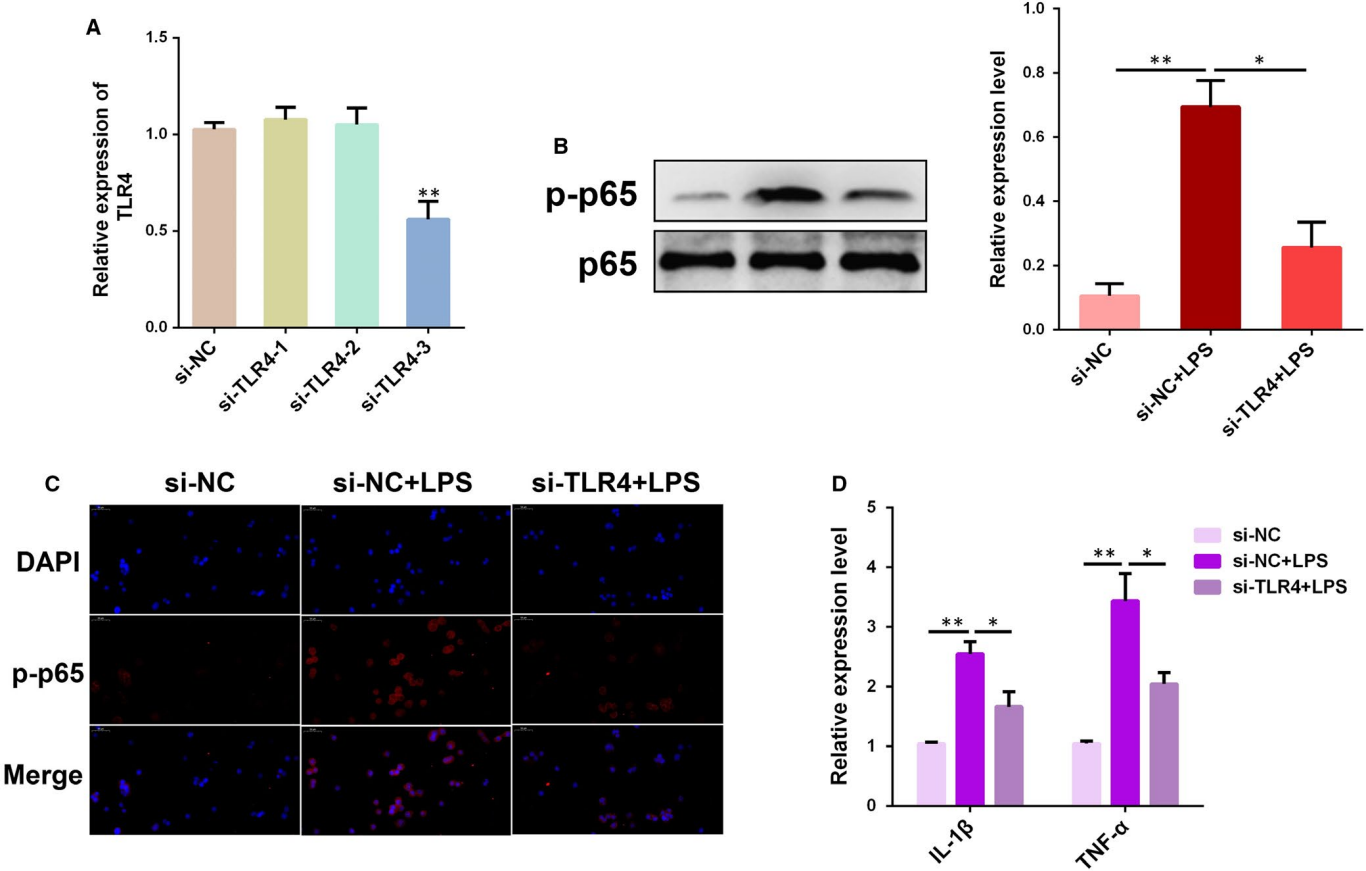
Knockdown of TLR4 attenuates LPS‐induced inflammatory response. A, BEND cells were transfected with TLR4 siRNA (si‐TLR4) or the negative control (si‐NC) for 24 h and then stimulated with LPS for 12 h. The TLR4 expression was determined by qPCR. B, The protein level of NF‐κB p65 was measured by Western blotting. C, Translocation of the NF‐κB p65 subunit from the cytoplasm into the nucleus was assessed by immunofluorescence staining. Blue spots represent cell nuclei, and red spots indicate p‐p65 staining. D, The mRNA levels of pro‐inflammatory cytokines IL‐1β and TNF‐α were detected by qPCR. Data are presented as the mean ± SEM. *P < .05; **P < .01

To further unravel the underlying mechanisms by which miR‐148a modulates LPS‐induced inflammation, a specific siRNA for TLR4 (si‐TLR4) was employed to block or inhibit the expression of TLR4 (Figure 5A). As displayed in Figure 5B, knockdown of TLR4 significantly reduced the phosphorylateion level of NF‐κB p65, accompanied by the inhibition of the translocation of NF‐κB p65 from the cytosol to the nucleus (Figure 5C). Furthermore, the expression levels of the pro‐inflammatory cytokines IL‐1β and TNF‐α were also inhibited following knockdown of TLR4 (Figure 5D), which is consistent with the effects of miR‐148a agomiR. Taken together, these findings strongly suggest that miR‐148a attenuates LPS‐induced inflammation through restraining the TLR4‐NF‐κB pathway.

### 3.6 Decreased miR‐148a expression is correlated with endometritis in mice

**Figure 6:**
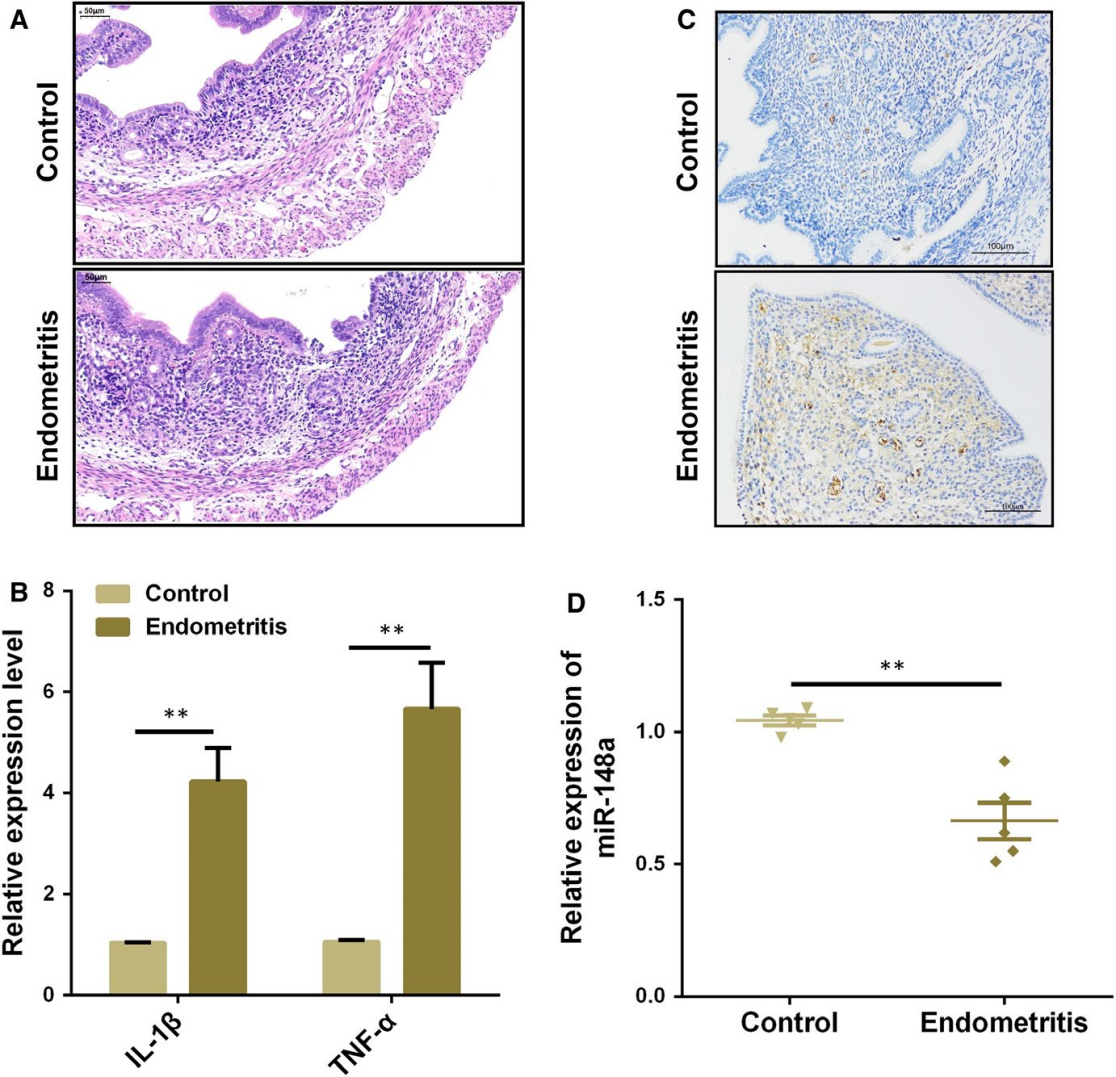
Expression of miR‐148a in the uterine tissues from mice with endometritis. A, H&E staining of uterine tissues. B, The mRNA levels of pro‐inflammatory cytokines IL‐1β and TNF‐α were detected by qPCR. C, Immunohistochemical staining of TLR4 in the uterine tissues from mice with endometritis. D, The miR‐148a expression in samples was measured by qPCR. Data are presented as the mean ± SEM. *P < .05; **P < .01

We induced endometritis in mice with LPS infusion and then performed H&E staining. As shown in Figure 6A, the control group showed a normal endometrial structure. However, the uterine tissues from the endometritis group displayed apparent pathological changes, including epithelial haemorrhage and inflammatory cell infiltration. The degree of inflammatory response in the endometrium was further evaluated by subsequent qPCR assay. The results of qPCR assay showed that the secretion of pro‐inflammatory cytokines IL‐1β and TNF‐α was markedly increased in the uterine tissues from mice with endometritis (Figure 6B). Our results also showed an increased level of TLR4 during endometritis (Figure 6C). We then determined the miR‐148a expression in the uterine tissues, and the expression of miR‐148a was dramatically repressed in the LPS group compared to the endometritis group (Figure 6D), revealing that aberrant miR‐148a expression is closely correlated with the pathogenesis of endometritis.

### 3.7 Delivery of miR‐148a agomiR alleviates LPS‐induced endometritis in mice

**Figure 7:**
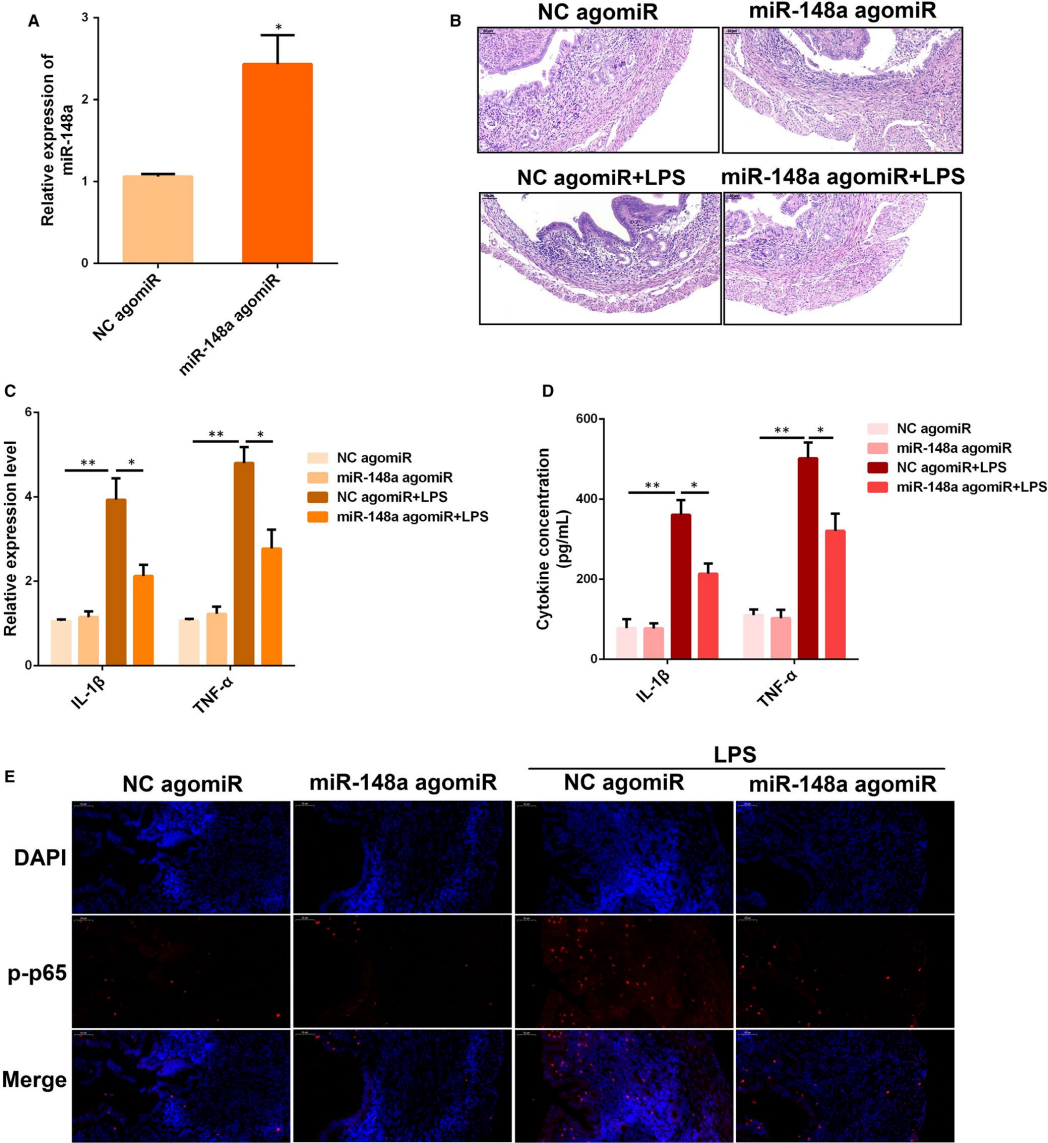
Delivery of miR‐148a agomiR alleviates experimental LPS‐induced endometritis in mice. (A) The miR‐148a expression was detected in uterine tissues by qPCR. (B) H&E staining of uterine tissues. The production of IL‐1β and TNF‐α was detected by qPCR (C) and ELISA (D). (E) Translocation of the NF‐κB p65 subunit from the cytoplasm into the nucleus was assessed by immunofluorescence staining. Blue spots represent cell nuclei, and red spots indicate p‐p65 staining. Data are presented as the mean ± SEM. *P < .05; **P < .01

Treatment with miR‐148a agomiR resulted in a marked increase in the uterine miR‐148a expression (Figure 7A), accompanied by the mitigated pathology conditions (Figure 7B). Moreover, miR‐148a agomiR also inhibited IL‐1β and TNF‐α expression (Figure 7C,D), as well as the NF‐κB p65 activation (Figure 7E). Overall, the results of the present study demonstrate the critical role of miR‐148a during endometritis and provide important new insights into the clinical implication of miR‐148a in the treatment of endometritis.

### 3.8 Inhibition of miR‐148a aggravates LPS‐induced endometritis in mice

**Figure 8:**
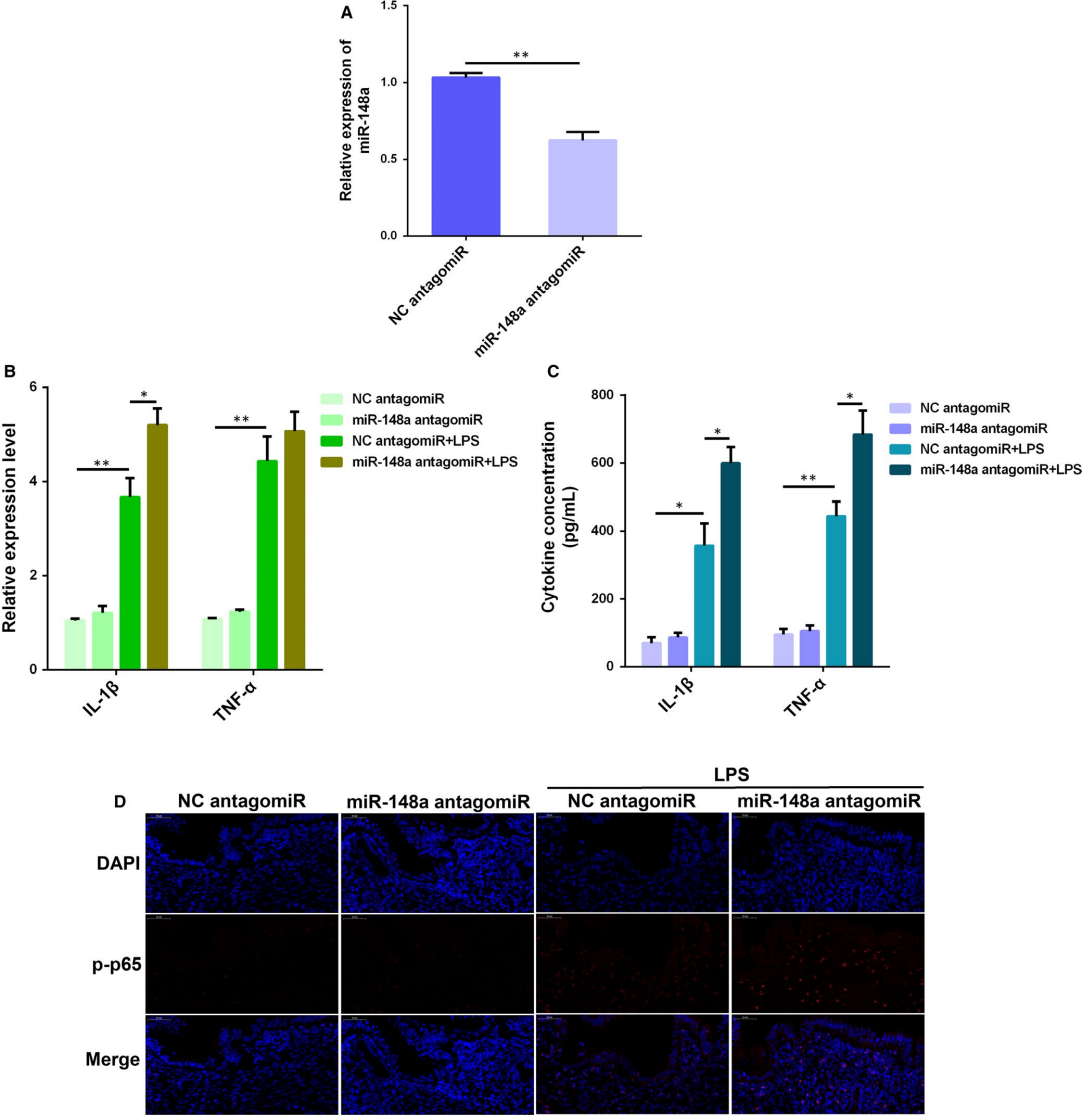
Inhibition of miR‐148a aggravates LPS‐induced endometritis in mice. (A) The miR‐148a expression was detected in uterine tissues by qPCR. The production of IL‐1β and TNF‐α was detected by qPCR (B) and ELISA (C). (D) Translocation of the NF‐κB p65 subunit from the cytoplasm into the nucleus was assessed by immunofluorescence staining. Blue spots represent cell nuclei, and red spots indicate p‐p65 staining. Data are presented as the mean ± SEM. *P < .05; **P < .01

The miR‐148a antagomiR was injected into mice before LPS treatment, leading to a significant suppression of miR‐148a in uterine tissues (Figure 8A). In addition, miR‐148a enhanced the activation of NF‐κB p65 (Figure 8D), and further increased the levels of IL‐1β and TNF‐α in uterus challenged with LPS, as examined by qPCR (Figure 8B) and ELISA (Figure 8C). These findings suggest that inhibition of miR‐148a aggravates LPS‐induced endometritis in mice.

## 4 DISCUSSION

It is quite important to elucidate the role of miRNAs in the pathogenesis of endometritis in dairy cows. Here, we observed that miR‐148a expression is significantly decreased in LPS‐stimulated endometrial epithelial cells. We then suggested for the first time that miR‐148a targets the 3′‐UTR of TLR4 mRNA and negatively regulate LPS‐induced NF‐κB p65 activation and pro‐inflammatory cytokines production. Furthermore, in vivo results also demonstrated that up‐regulation of miR‐148a using synthetic agomiR alleviates the inflammatory conditions in the uterine tissues of LPS‐challenged mice.

Amongst the common diseases of dairy cows, bacterial infections of reproductive tract have a tremendous impact on the dairy industry.^5^ The uterus is the reproductive tract of mammals and is susceptible to bacterial infections after partum, especially for high‐producing cows.^32^, ^33^ The uterine lumen of postpartum cows usually contains diverse species of bacteria, but clinical diseases (such as endometritis) do not always occur. The establishment and development of endometritis depend partly on the balance of host defensive response and bacterial species.^3^, ^34^ Indeed, in the process of bacterial invasion into the uterus, the endometrium reacts to foreign pathogens by affecting immune response‐related signalling pathways.^35^ Hence, understanding how these molecular events are regulated will contribute to identify molecular markers that can be used as indicators of endometritis. It has been shown that uterus is a dynamic reproductive organ with strict gene regulation at transcriptional and post‐transcriptional levels.^36^ Abnormal miRNAs expression is associated with the occurrence of various functional disorders in bovine uterus.^37^ Specifically, the important roles of miRNAs in the development of reproductive systems in ruminants, such as cattle, have been widely recognized in previous studies.^38^ miR‐29a, which was selectively expressed by the follicular cells, has been confirmed to be involved in gene regulation during early phase of corpus luteum (CL) development.^39^ Similarly, some miRNAs, such as let‐7f and miR‐125b, participate in the regulation of bovine cyclic reproductive activity.^40^ Tscherner et al^41^ reported that miR‐34c may have the potential as a non‐invasive and quantifiable biomarker for assessing the reproductive capacity of cattle. miR‐148a, belongs to the miR‐148/152 family, is a highly conservative miRNA in mammals. miR‐148a has been reported to be critical for regulating tumour growth, inflammation and immunity.^42^, ^43^ More importantly, an analysis of miRNA sequencing data showed that miR‐148a was differentially expressed between the healthy and endometritis cows.^27^ Another study indicated that miR‐148a expression was altered in bovine mammary epithelial cells challenged with *E coli*, and miR‐148a was unique to *E coli* infection.^44^ Indeed, miR‐148a could inhibit DSS‐induced colitis in mice.^28^ Interestingly, our present study also found that miR‐148a expression was dramatically reduced in LPS‐stimulated BEND cells, further suggesting that miR‐148a may possess an imperative role in the pathogenesis of endometritis.

It is a well accepted fact that the initial defence of endometrium against microbial infection and tissue damage mainly depends on the innate immune system of the organism.^9^, ^45^ Innate immunity of endometrium is a non‐specific protection, including physical barrier and secretory protein.^14^ For example, an anatomical barrier consisting of the cervix prevents bacteria from entering the uterine cavity. Some mucosal glycoproteins can also cover the mucosa to neutralize bacteria and prevent bacteria from invading the epithelium.^46^ In addition, TLRs are another important component of innate immunity.^47^ LPS from *E coli* is a known exogenous ligand for TLR4, which strongly initiates a remarkable and persistent inflammatory response that is featured by the secretion of chemokines and cytokines and the disruption of epithelial integrity.^48^ In the present work, we noticed that overexpression of miR‐148a significantly repressed the levels of pro‐inflammatory cytokines IL‐1β and TNF‐α in LPS‐stimulated BEND cells. Moreover, activation of NF‐κB promotes the transcription of genes of inflammatory mediators, which is responsible for the initiation and progression of endometritis.^49^ Thus, to better understand the molecular mechanisms through which miR‐148a reduces the pro‐inflammatory cytokines production, we further determined the effect of miR‐148a on NF‐κB activation. As expected, the phosphorylation level of NF‐κB p65 by LPS was notably suppressed after miR‐148a overexpression. These findings suggest that miR‐148a suppresses LPS‐induced inflammatory response, possibly by restraining the activation of NF‐κB pathway.

It is widely recognized that activated TLR4 recruits its downstream adaptor molecules, such as MyD88, IRAK1 and TRAF6, and then leads to activation of several transcription factors such as NF‐κB.^50^ A substantial body of research has revealed that TLR4 has a tight relationship with many diseases. For example, knockout of TLR4 could suppress the pro‐inflammatory state of diabetes in mice.^51^ TLR4 knockout mice have neuroprotective effects against ischaemia/reperfusion‐induced brain injury.^52^ Moreover, previous studies have also reported that the expression of TLR4 in the endometrium of postpartum cattle that develop endometritis and infertility was apparently higher than that of normal cattle.^53^ Based on these findings, we suspected that reduction of TLR4 expression could attenuate the inflammatory condition of endometritis. Here, we observed that overexpression of miR‐148a markedly decreased the protein levels of TLR4, MyD88, IRAK1 and TRAF6 in LPS‐stimulated BEND cells. On the other hand, bioinformatics predictions performed using available miRNA databases (such as TargetScan 7.2) implied that TLR4 is a potential molecular target of miR‐148a. To test the prediction, the luciferase reporter assay was carried out in subsequent studies. A notable decrease of luciferase activity upon miR‐148a agomiR transfection was found, manifesting that TLR4 is a actual target molecule of miR‐148a. In fact, some bovine miRNAs, such as miR‐19a, have been shown to negatively regulate the TLR4‐mediated inflammatory reaction in vitro.^54^ Overally, these results strongly suggested miR‐148a as an important negative regulator of TLR4, leading to the attenuation of LPS‐elicited inflammatory response.

A murine model of LPS‐induced endometritis has been confirmed to be a useful model for evaluating the therapeutic strategies against endometritis.^55^ Thus, to further explore whether miR‐148a could act as a potential target for the treatment of endometritis, an experimental mouse endometritis model was created by LPS infusion. Intriguingly, overexpression of miR‐148a using agomiR significantly alleviated the uterine injury in mice, as evidenced by the attenuated pathological conditions; while inhibition of miR‐148a using antagomiR had inverse effects. Besides, miR‐148a agomiR also decreased the pro‐inflammatory cytokines production and increased the repression of NF‐κB activation in vivo, which is consistent with the in vitro results.

In conclusion, our present study identifies for the first time that miR‐148a functions as a negative regulator of the inflammatory response in LPS‐induced endometritis by targeting TLR4 and its downstream pathway. Therefore, pharmacologic stabilization of miR‐148a may serve as an effective strategy in treating endometritis and other inflammation‐related diseases.

## CONFLICT OF INTEREST

The authors have declared no competing financial interest.

## AUTHOR'S CONTRIBUTION

KJ and GD designed this study. KJ, JY, CY, TZ and HW performed the experiments. KJ, JY, TZ, AS, AD and XY analysed the data. KJ, JY and GD wrote the manuscript. All authors have read and approved the final manuscript.

## Data Availability

The data that support the findings of this study are available from the corresponding author upon reasonable request.
