# Supplementary figures and images for: miR‐148a suppresses inflammation in lipopolysaccharide‐induced endometritis
Source: J Cell Mol Med. 2019 Nov 22;24(1):405–17. doi: 10.1111/jcmm.14744 (PMC6933404; doi:10.1111/jcmm.14744)

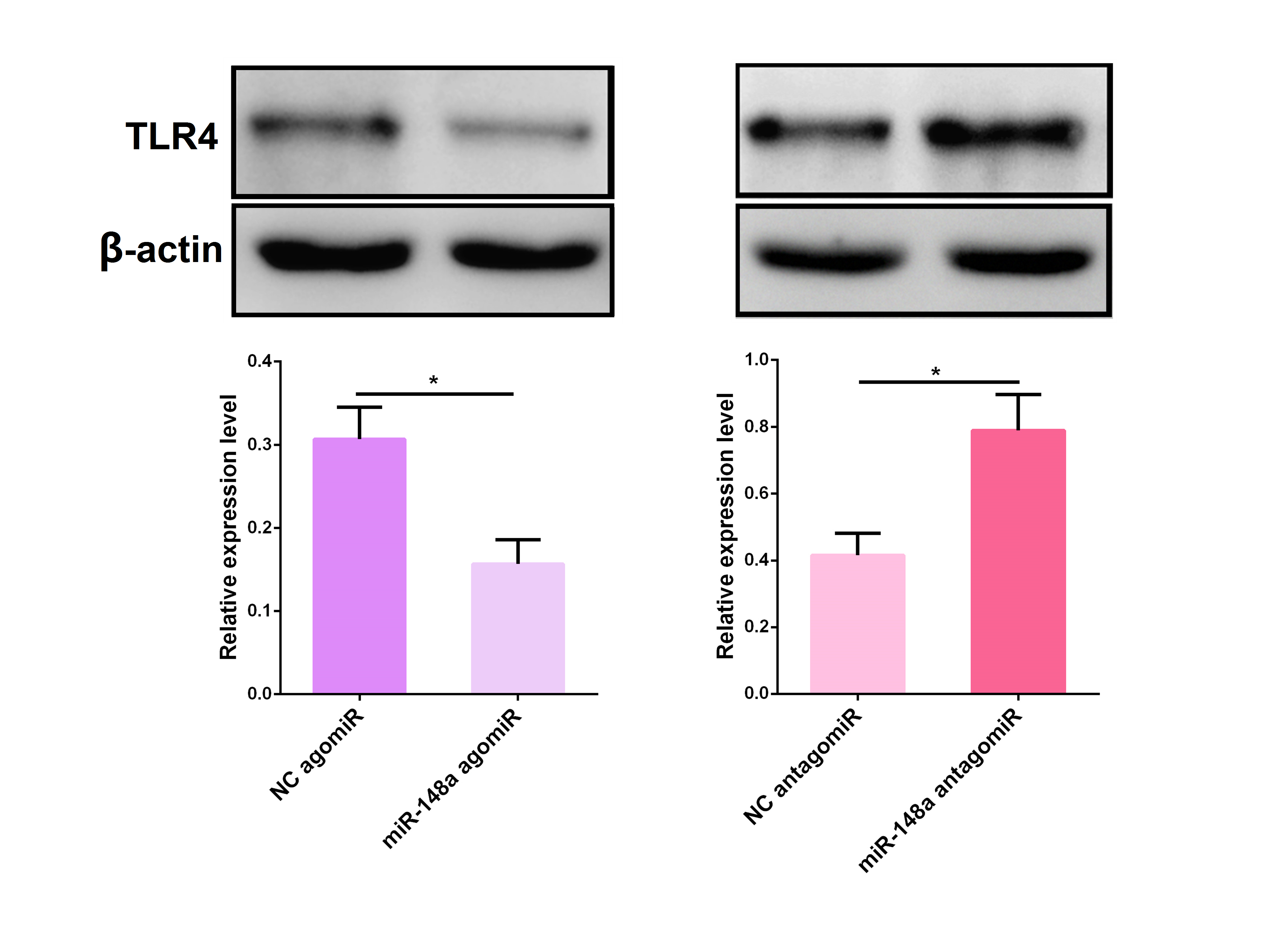

Supplement: Supplementary file 1 [file JCMM-24-405-s001.tif]
