# Supplementary material for: miR‐148a suppresses inflammation in lipopolysaccharide‐induced endometritis
Source: J Cell Mol Med. 2019 Nov 22;24(1):405–17. doi: 10.1111/jcmm.14744 (PMC6933404; doi:10.1111/jcmm.14744)
Supplement: Supplementary file 2 [file JCMM-24-405-s002.docx]

**Figure S1. miR-148a inhibits TLR4 expression *in vitro*.** BEND cells were transfected with miR-148a agomiR, miR-148a antagomiR, or the corresponding negative controls (NC agomiR, NC antagomiR), and the TLR4 protein levels were measured by Western blotting. Data are presented as the mean ± SEM. **P* < 0.05; ***P* < 0.01.
